# JMJD6 is a driver of cellular proliferation and motility and a marker of poor prognosis in breast cancer

**DOI:** 10.1186/bcr3200

**Published:** 2012-05-23

**Authors:** Yi Fang Lee, Lance David Miller, Xiu Bin Chan, Michael A Black, Brendan Pang, Chee Wee Ong, Manuel Salto-Tellez, Edison T Liu, Kartiki V Desai

**Affiliations:** 1 Department of Cancer Biology and Pharmacology, Genome Institute of Singapore, 60 Biopolis Street, Genome Building, 138672, Singapore; 2 Department of Cancer Biology, Wake Forest University School of Medicine, Winston-Salem, NC 27157, USA; 3 Department of Pathology, National University Health System and National University of Singapore, 5 Lower Kent Ridge Road, 119074, Singapore; 4 Centre for Cancer Research and Cell Biology, Queen's University Belfast, 97 Lisburn Road, Belfast, BT9 7BL, UK; 5 Cancer Science Institute, National University of Singapore, 28 Medical Drive, 117456, Singapore; 6 Department of Biochemistry, Otago School of Medical Sciences, University of Otago, 710 Cumberland Street, Dunedin 9054, New Zealand; 7 The Jackson Laboratory, 600 Main Street, Bar Harbor, ME 04609, USA; 8 National Institute of Biomedical Genomics, 2nd Floor Netaji Subash Sanatorium, Kalyani 741251, India

## Abstract

**Introduction:**

We developed an analytic strategy that correlates gene expression and clinical outcomes as a means to identify novel candidate oncogenes operative in breast cancer. This analysis, followed by functional characterization, resulted in the identification of Jumonji Domain Containing 6 (JMJD6) protein as a novel driver of oncogenic properties in breast cancer.

**Methods:**

Through microarray informatics, Cox proportional hazards regression was used to analyze the correlation between gene expression and distant metastasis-free survival (DMFS) of patients in 14 independent breast cancer cohorts. *JMJD6 *emerged as a top candidate gene robustly associated with poor patient survival. Immunohistochemistry, siRNA-mediated silencing, and forced overexpression of JMJD6 in cell-based assays elucidated molecular mechanisms of JMJD6 action in breast cancer progression and shed light on the clinical breast cancer subtypes relevant to JMJD6 action.

**Results:**

*JMJD6 *was expressed at highest levels in tumors associated with worse outcomes, including ER- and basal-like, Claudin-low, Her2-enriched, and ER^+ ^Luminal B tumors. High nuclear JMJD6 protein was associated with ER negativity, advanced grade, and poor differentiation in tissue microarrays. Separation of ER^+^/LN^- ^patients that received endocrine monotherapy indicated that *JMJD6 *is predictive of poor outcome in treatment-specific subgroups. In breast cancer cell lines, loss of *JMJD6 *consistently resulted in suppressed proliferation but not apoptosis, whereas forced stable overexpression increased growth. In addition, knockdown of *JMJD6 *in invasive cell lines, such as MDA-MB231, decreased motility and invasion, whereas overexpression in MCF-7 cells slightly promoted motility but did not confer invasive growth. Microarray analysis showed that the most significant transcriptional changes occurred in cell-proliferation genes and genes of the TGF-β tumor-suppressor pathway. High proliferation was characterized by constitutively high cyclin E protein levels. The inverse relation of *JMJD6 *expression with *TGF-β_2 _*could be extrapolated to the breast cancer cohorts, suggesting that *JMJD6 *may affect similar pathways in primary breast cancer.

**Conclusions:**

*JMJD6 *is a novel biomarker of tumor aggressiveness with functional implications in breast cancer growth and migration.

## Introduction

In breast cancer, resistance to standard-of-care systemic adjuvant treatments such as endocrine and chemotherapies remains a major health burden and prompts the need for novel therapeutic targets for patients with advanced, unresponsive, or relapsed disease. We previously used gene-expression profiles of breast tumors to identify extracellular/secretory proteins and cell surface-receptor genes whose high expression levels associate with poor clinical end points. For example, we recently identified serine protease inhibitor Kazal-type 1 (*SPINK1*) as an important therapeutic target in breast cancer by using a combined genotype and phenotype screening approach. We found that inhibition of SPINK1 by neutralizing antibodies curtailed multiple aggressive properties, including cell survival, invasiveness, and chemoresistance [1]. A second candidate identified in the same study was the phosphatidylserine receptor (*PTDSR*).

Formerly, PTDSR was thought to be a cell-surface protein that facilitates recruitment of phagocytic cells to sites of apoptosis. Antibodies against PTDSR and annexin V have been used in combination to estimate apoptosis [2]. Mouse knockouts of PTDSR showed early postnatal lethality and had growth retardation and multiple developmental abnormalities due to insufficient differentiation during embryogenesis; however, no defect in apoptotic clearance of cells was evident [3]. By generation of deletion mutants and immune localization, Cui *et al. *[4] demonstrated that PTDSR is a nuclear protein, with five nuclear localization signals scattered throughout its sequence. Later, PTDSR was renamed Jumonji domain containing 6 (*JMJD6*) based on the presence of its JMJC domain with bifunctional histone arginine demethylation and lysyl oxidase activity [4-6]. JMJD6 is homologous to the hypoxia-inducible factor (HIF) asparaginyl-hydroxylase, suggesting a function in cellular response to hypoxia. In addition, JMJD6 protein was recently shown to interact with splicing factor U2AF65; however, very few splicing events in a limited number of genes were attributable to *JMJD6 *expression [6]. In endothelial cells, alternate splicing of VEGF receptor (Flt1) by U2AF65 promoted endothelial cell migration, and siRNA-mediated knockdown of *JMJD6 *in endothelial cells led to decreased migration [7]. Based on X-ray crystallographic data, it was predicted and shown that apart from its enzymatic activity, JMJD6 protein bound single-stranded RNA [8]. These diverse findings predict a range of versatile functions for JMJD6, at the transcriptional, splicing, posttranscriptional, and biochemical levels. However, very little is known about the role of JMJD6 in cancer and the molecular pathways that may impinge on disease initiation and prognosis.

Because our *in silico *analysis demonstrated a robust positive association between *JMJD6 *expression and breast cancer recurrence, we investigated its phenotypic and molecular effects in breast cancer cells. We report herein that perturbation of *JMJD6 *expression modulates cell proliferation and cell scattering and motility: phenotypes associated with cancer metastasis. Furthermore, our findings suggest that these cellular phenotypes may be elicited by JMJD6-mediated suppression of transforming growth factor-beta 2 (TGF-β2) and/or activation of proteins that potentiate cell division in a cell type-specific manner. These *in vitro *mechanistic findings are consistent with the clinical observations that *JMJD6 *expression correlates positively with proliferation index and high histologic grade but inversely with *TGF-β2 *expression. Together, these data implicate JMJD6 function in breast tumor progression and suggest a diagnostic role for JMJD6 in predicting patient outcomes.

## Materials and methods

### Breast cancer clinical datasets

Tumor-expression profiles were obtained with approval from and in accordance with the policies of the institutional review boards of the respective institutions. An integrated "Super Cohort" (SC) of 15 individual Affymetrix array datasets comprising 2,116 breast cancer patients was used in this analysis. These datasets were previously described in detail in Soon *et al. *[1]. These cohorts were accessible from public databases, Gene Expression Omnibus (National Center for Biotechnology Information, Bethesda, MD, USA), ArrayExpress (European Bioinformatics Institute, Hinxton, UK), and caArray (National Cancer Institute, NIH, Atlanta, GA, USA). Appropriate permission has been granted for the use of the datasets and corresponding de-identified clinical data. A summary of the clinical dataset details and literature references can be found in Additional file 1, Table S1. Initial discovery and meta-analysis was performed on a subset of this Super Cohort.

All raw data (CEL files) were preprocessed and normalized by using the R software package [9], and library files provided via the Bioconductor [10]. Raw data were MAS5.0 normalized on a per-cohort basis by using the justMAS function in the simpleaffy library from Bioconductor (no background correction, target intensity of 600). Cross-cohort batch effects were corrected by using the COMBAT empirical Bayes method [11]. Normalized *JMJD6 *probesets, 212722_s_at and 212723_at, were averaged for the analysis of data from Affymetrix U133 arrays, and *JMJD6 *probe, AB011157, was used for the analysis of the Agilent array dataset (that is, the NKI dataset). Of 2,116 array profiles in the Super Cohort, 1,954 patient cases are annotated with distant metastasis-free survival (DMFS) time and event information.

### Clinical survival analysis and expression in subtypes

Distant metastasis-free survival (DMFS) was used as the clinical end point of interest. A DMFS event was defined as metastatic recurrence to a distant organ site or in a limited number of cases, as death owing to progressive breast cancer. Cox proportional hazard regression analysis of *JMJD6 *expression with DMFS data (Time and Event) was performed in individual patient datasets and in the Super Cohort. Clinical subtype analysis was performed by using the Super Cohort. Intrinsic subtypes were assigned via the PAM50 algorithm of Parker *et al. *[12], by using the code provided by the authors at UNC Microarray Database [13]. Gene data were matched by symbol and median centered, and Spearman correlation was used to assign samples to the nearest PAM50 centroid. Claudin-Low subtypes were assigned based on the method described by Prat *et al. *[14], by using the microarray data (GSE18229) and information provided by the authors at UNC Microarray Database [13]. Claudin-Low and Normal centroids were generated, and samples were assigned to one or the other class based on euclidean distance to the class centroid. The PAM50 and Claudin-Low subtype information was then combined with the PAM50 subtype used, unless a sample had been classified as Claudin-Low, in which case, the Claudin-Low assignment would take precedence.

Distribution of *JMJD6 *expression in various breast cancer subtypes was analyzed with the Kruskal-Wallis one-way analysis of variance (ANOVA) on ranks and multiple pairwise comparisons with the Dunn method by using Sigma Plot. Statistical significance of differential *JMJD6 *expression in ER-positive versus ER-negative tumors was analyzed by using the Mann-Whitney Rank Sum Test. In Kaplan-Meier and CoxPH survival analysis of *JMJD6 *expression cohorts in the clinical subtypes, the patients were separated by median expression across the Super Cohort into high and low *JMJD6*-expression groups. Kaplan-Meier survival analysis also was performed on patient groups ranked by quartile expression within each clinical subtype. All survival analyses were performed by using Sigma Plot [15].

### Immunohistochemistry

Tissue microarray (TMA) blocks containing cores from 98 breast cancer patients were constructed, as described previously, under institutional ethics committee approval with consent for the tissue microarray program (NUS-IRB 05-017) [1,16,17] and used for the analysis. Anti-JMJD6 monoclonal antibody (Santa Cruz, Santa Cruz, CA, USA: Sc-28348) was used at a dilution of 1:50 along with antigen retrieval by heat and Tris-EDTA (pH 9.1). Automated IHC scoring was performed with the Ariol SL-50 image analysis system (Applied Imaging, Santa Clara, CA, USA). Positivity of JMJD6 nuclear expression was defined as nuclear-staining intensity and percentage coverage scores ≥25%. Odds-ratio analysis was performed on JMJD6-positive expression and clinicopathologic features of the tumors by using PASW Statistics 18 [18].

### Cell lines

All cell lines were obtained from American Type Culture Collection (ATCC) and maintained in growth media, at 37°C with 5% CO_2_. The growth medium for MCF-7, CAMA-1, and BT-549 was Dulbecco Modified Eagle Medium (DMEM), with 10% fetal bovine serum (FBS); and for MDA-MB231 and T47D, it was RPMI-1640, with 10% FBS.

### Cloning and expression of *JMJD6 *

*JMJD6 *(NM_001081461.1) (J1) was amplified by using MCF-7 mRNA (forward primer: 5'CCCAAGCTTATGAACCACAAGAGCAAGAAG3'; reverse primer: 5' GCTCTAGATCACCTGGAGGAGCTGCG 3'), followed by reamplification with forward primer: 5' GAGGTACCATGAACCACAAGAGCA 3' and reverse primer: 5' CGCTCGAGTGGGGGTGAGCCCGGCCT 3' and ligated into TOPO pCR2.1 vector. All clones were sequence verified. *JMJD6 *was cloned from TOPO vectors into a gateway entry vector pENTR3C (Invitrogen) and then recombined into the lentivirus vector pLenti6.2/V5-DEST (Invitrogen), pLenti4/V5-Dest (Invitrogen), and pcDNA3.1/V5-Dest (Invitrogen) by LR recombination, according to the manufacturer's protocol. For lentiviral clones, pLenti6.2/V5-DEST or pLenti4/V5-Dest was co-transfected with packaging vectors (Invitrogen) into 293 FT cells, and the supernatant was harvested approximately 48 hours for packaged lentivirus. For infection of MCF-7 cells, the cells were plated at 50% confluence and incubated with 20 μl to 30 μl of the concentrated virus and 8 μg/ml of hexamethrine bromide (Polybrene) for 24 hours at 37°C. The cells were replated and cultured with Zeocin (Invitrogen) to obtain several clones (MCF7-J1-OE). The pcDNA3.1 vector was used to generate *JMJD6 *expression clones by transfection by using Lipofectamine 2000 (Invitrogen) into MDA-MB231, T47D, and CAMA cell lines. Stable clones were selected with gentamycin (Gibco).

### Knockdown of *JMJD6 *gene

SiRNA was reverse transfected (by using manufacturer's protocol) by using Lipofectamine 2000 (Invitrogen) into the cell lines. At 48 hours after transfection, the cells were reconstituted in fresh media for experimental assays. *JMJD6 *siRNAs used were as follows: siRNA A (Ambion: 111915)- gcuauggugaacacccuaatt; siRNA B (Dharmacon: D-010363-01)- gaacugggauucacaucga; siRNA C (Dharmacon: D-010363-02)- ggauaacgauggcuacuca; and siRNA D (Dharmacon: D- 010363-05)- ggacccggcacaacuacua. A nontargeting scrambled siRNA served as a negative control (Ambion: 4635).

### Cell-based assays

For proliferation assay, cells were plated in 96-well plates at a density of 5,000 cells per well. Cell proliferation was measured by using WST-1 (Roche) every 24 hours over a 4- to 5-consecutive-day period, according to manufacturer's protocol. For detecting apoptosis, cells were transfected in a 96-well plate with siRNAs and assessed for apoptotic markers after 48 hours. In brief, the cells were fixed with paraformaldehyde, and cells were permeabilized with Triton-X 100. Fixed cells were probed with active PARP (BD, 552596) antibody, and the primary antibodies were detected by a secondary antibody conjugated to fluors Alexa 488 (BD, A21121). ArrayScan VTI (Cellomics) was used to detect immunofluorescence, and the baseline threshold was set by using cells stained with secondary antibody alone in the absence of the primary antibody.

### Scatter assay

Cells were plated in six-well plates at a density of 300 cells per well and grown in DMEM with 10% FBS. Fifteen fields of colonies per cell type were captured on day 7 of growth and blindly scored for three categories of scattering (compact, loose, and scatter) by two random individuals from six people. An average score was calculated for each colony, and the percentage of colonies for each category of scattering was plotted [19].

### Wound-healing assay

Cells were plated in culture inserts (Ibidi) with approximately 500 μ*M *gap. After 2 days (confluent cells), the inserts were removed, and fresh medium with 5 μg/ml of mitomycin C (Calbiochem, 47589) was added. The distances moved by the cells across the gap were measured at 24 hours and calculated as a ratio over the initial distance at 0 hours, and these data were further normalized to the ratio of distance in Vec control.

### Invasion and migration assay

*In vitro *migration and invasion of the cells were assessed by using Falcon FluoroBlok 24-Multiwell inserts with 8-μm pores (BD Biosciences). For invasion assays, the inserts were coated with 20 μg of Matrigel in 80 μl of serum-free media. Cell suspension (200 μl) in serum-free media was loaded into each transwell insert, and 750 μl of medium with 10% FBS was provided in the lower chamber; 4 × 10^4 ^MDA-MB231/BT-549 cells were loaded in each transwell. The assay was done for 18 hours for migration and 24 hours for invasion. The cells that had migrated or invaded through the inserts were fixed with 3.7% formaldehyde and stained with propidium iodide, 2 μg/ml (Calbiochem), for 30 minutes, washed with PBS, and counted for 10 fields by using the Target Activation Bioapplication on an ArrayScan VTI (Cellomics). Assay results for siRNA-treated MDA-MB231 cells were normalized to fold change observed in proliferation at Day 2 and then calculated as a ratio to the Sc siRNA control.

### Microarray data analysis

MCF-7 J1 clones were harvested for RNA isolation at 80% confluence. For siRNA-mediated knockdown of *JMJD6 *in MCF-7 and MDA-MB231, the cells were harvested for RNA isolation immediately, 48 hours after transfection. Cells were washed twice with PBS and harvested by trypsinization. RNA was extracted by Trizol (Invitrogen) according to manufacturer's protocol. Three biological replicates of MCF-7 J1 clones and two independent siRNA-transfection replicates were used for the microarray hybridization. Processing of samples for hybridization on Affymetrix U133 Plus 2.0 was done according to manufacturer's protocol. The microarray data were uploaded to Gene Expression Omnibus (accession number, GSE31782). Raw data were MAS5-normalized and log transformed by using Genespring GX11.5. To extract differential gene expression, GeneSpring GX 11.5 was used to perform two-way ANOVA for siRNA-specific changes in MCF-7 and MDA-MB231. The list of significantly differential genes (*P *< 0.05) was further filtered for genes with at least 1.5-fold change in expression in at least one of the siRNA treatments for either MCF-7 or MDA-MB231. For MCF-7 J1-OE clones, one-way ANOVA was performed to extract differential gene expression in the overexpression clones as compared with the Vec. Only genes that differed in their expression by 1.5-fold in at least one of the clones were selected for further analysis. Genes that overlapped in both siRNA treatment and in J1 clones and those that were regulated in the opposite direction were selected for Ingenuity Pathway Analysis (IPA) [20]. Hierarchical clustering by average linkage and visualization of the clusters was performed by using Cluster and Treeview, respectively [21,22].

To extract differential gene expression specific to MDA-MB231, GeneSpring GX11.5 was used to perform one-way ANOVA in MDA-MB231 and MCF-7 independently. The list of significant genes that were differentially expressed (1.5-fold; *P *< 0.05) was filtered. Probesets unique to MDA-MB231 that were absent in MCF-7 lists were extracted for IPA functional analysis.

### Real-time PCR

Total RNA was reverse transcribed by using Maxima reverse transcriptase mix (Fermentas). Real-time PCR was performed by using SYBR-Green PCR Mix (Fermentas) and run on CFX384 Real Time PCR Detection System (Biorad). Ct values were generated by using CFX manager software (Biorad). The primers used are listed in the Additional file 2, Table S2.

### Conditioned media

To obtain conditioned media, plated cells were incubated with serum-free media for 24 hours before collection. The media were spun down to remove any cell debris and then concentrated ×60 to 80 by using Amicon Ultra centrifugation filter (Millipore).

### Immunoblot analysis

Whole-cell lysates were prepared by using RIPA buffer for all antibodies, except TGF-βs, detected in conditioned media. Protein concentration was quantified by using Protein Assay reagent (Biorad) with BSA standards. Equal amounts of total protein lysates (20 to 40 μg) were analyzed on an SDS-PAGE gel and transferred to PVDF membrane (GE Healthcare and Millipore). Antibodies against JMJD6 (Abcam, ab10526), β-actin (Sigma, A5441), V5 tag (Invitrogen, R960-25), E-cadherin (Cell Signaling, 4065), vimentin (BD Pharmingen, 550513), TGF-β2 (Abcam, ab36495), phosphorylated SMAD2 (Cell Signaling, 3108 and 3104), phosphorylated SMAD3 (Cell Signaling, 4520), SMAD2/3 (Cell Signaling, 3102), cyclin D1 (ab24249), cyclin E1 (Abcam, ab3927), and cyclin E2 (Abcam, ab40890) were used to probe for the protein on the membrane. The detection was done by using HRP-conjugated antibodies (Santa Cruz) and ECL or ECL Plus reagents (Amersham Biosciences). Reactive proteins were identified with autoradiography.

### TGF-β2 neutralization and Smad phosphorylation assays

Cells were plated in 96-well plates at a density of 5,000 cells per well and changed to fresh media with either 5 ng/ml of recombinant human (rh) TGF-β2 (R&D Systems, 302-B2) or BSA the next day. Cell-viability measurement was taken on the first day of TGF-β2 or BSA treatment and 3 days later by using WST-1 assay. To inhibit TGF-β2 action, cells were plated in 96-well plates at a density of 5,000 cells per well. After 24 hours, they were exposed to 10 μ*M *SB431542 (Sigma-Aldrich, S4317) or DMSO (as vehicle control) before transfection with JMJD6 siRNA. The cells were changed to fresh media 48 hours after transfection, containing 10 μ*M *SB431542 or DMSO. Cell viability was determined on the first day of SB431542 or DMSO treatment and 3 days later by WST-1 assay. For assessment of SMAD2 phosphorylation, cells were plated at approximately 50% confluence in six-well plates for 24 hours. The cells were pretreated for 2 hours with SB431542 (Sigma-Aldrich, S4317) or DMSO, before addition of 5 ng/ml of rhTGF-β2 (R&D Systems, 302-B2). Protein lysates were harvested 1 hour after rhTGF-β2 treatment for immunoblot analysis.

### Clinical correlation between *TGF-β2 *and *JMJD6 *

The super-cohort dataset was used to assess correlation, if any, between *JMJD6 *and *TGF-β2 *expression. *TGF-β2 *probesets, 209908_s_at, 220407_s_at, 209909_s_at, and 220406_at, were averaged for the clinical microarray data analysis. Correlation of *JMJD6 *expression and *TGF-β2 *expression was performed by using the Pearson correlation test on PASW Statistics 18 [18].

## Results

### *JMJD6 *transcript levels correlate with poor prognosis in breast cancer

**Figure 1:**
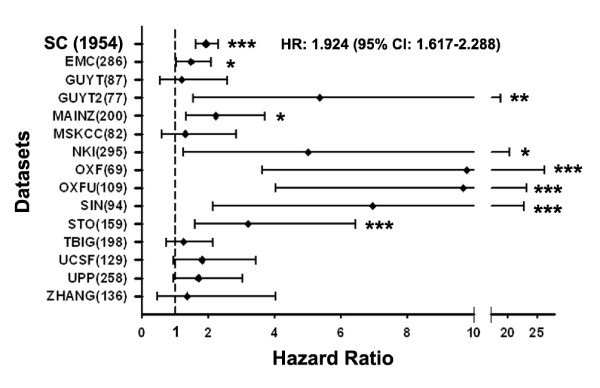
**High *JMJD6 *mRNA expression is associated with poor survival outcome**. Cox regression analysis of *JMJD6 *expression and distant metastasis-free survival of patients was initially performed in 14 microarray expression datasets independently and then subsequently on a "Super Cohort" (SC) comprising 15 Affymetrix array-based cohorts, including a subset of the 14 cohorts used initially. Hazard ratios and their 95% confidence intervals are shown in the forest plot. Datasets are listed on the Y-axis; all platforms are from Affymetrix array series, with the exception of NKI from Agilent. The 95% confidence intervals are indicated by the error bars, and the cohort size is shown in parentheses. *P ≤ 0.05; **P ≤ 0.01; ***P ≤ 0.005.

To discover novel oncogenes associated with breast cancer progression, we used an informatics approach [1] and mined multiple independent microarray datasets of breast tumor profiles for genes having significant and reproducible associations with distant metastasis-free survival (DMFS). *JMJD6 *emerged as a top candidate, because its expression was significantly and positively associated with decreased time to distant metastasis in eight of the 14 breast cancer cohorts examined (Figure 1 and Additional file 1, Table S1). Furthermore, in an integrated Super Cohort (SC) (*n *= 1,954) comprising the majority of the individual cohorts listed in Table 1 (see Methods and Additional file 1, Table S1), high *JMJD6 *expression was significantly associated with decreased time to distant metastasis with a hazard ratio of 1.92 (95% CI, 1.62 to 2.29; *P *< 0.001). Together, these observations indicate a robust and reproducible association between *JMJD6 *expression and aggressive clinical behavior of breast cancer that may reflect an underlying functional contribution of *JMJD6 *in breast cancer progression.

**Table 1:** Hazard ratios of *JMJD6 *expression groups in various subtypes of breast cancer

Subtypes	Cohort size (*JMJD6 *high, low)	Hazard ratio	(95% CI)	*P *value
Basal	84, 250	0.904	0.572-1.43	0.667
Claudin-Low	19, 73	1.577	0.542-4.587	0.403
Her2	98, 183	0.843	0.56-1.27	0.415
**Lum A**	309, 256	**1.524**	**1.009-2.304**	**0.045**
**Lum B**	116, 283	**1.712**	**1.132-2.591**	**0.011**
ER ^-^	247, 154	1.067	0.742-1.536	0.725
**ER^+^**	731, 822	**1.912**	**1.546-2.364**	**< 0.001**

CoxPH analysis of *JMJD6 *expression cohorts (high versus low separated by median of *JMJD6 *expression across the Super Cohort) was performed in annotated subtypes of breast cancer. The table shows the number of patients used for the analysis in the high- and low-*JMJD6 *expression cohorts, the hazard ratio, and its 95% confidence interval (CI) and *P *value generated from the analysis in each subtype.

### *JMJD6 *expression in breast cancer subtypes

**Figure 2:**
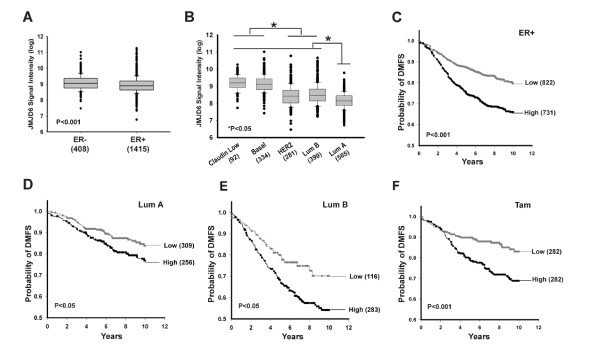
***JMJD6 *expression and prognosis in intrinsic subtypes of breast cancer**. Box plots of *JMJD6 *expression demonstrate **(A) **higher *JMJD6 *probeset intensity (log2) in ER^- ^tumors compared with ER^+ ^tumors, with *P *< 0.001 by Mann-Whitney Rank Sum test; and **(B) **highest *JMJD6 *probeset intensity (log2) in claudin-low and basal subtypes, followed by *HER2*-enriched and LumB subtypes, and lowest in LumA subtypes. The Dunn method was used to perform pairwise multiple comparison among the subtypes. *P < 0.05 between a pair of subtypes. Dots of boxplots (A, B) represent outliers in the 90^th ^and 10^th ^percentiles. Kaplan-Meier survival curves based on below-median (low) and above-median (high) *JMJD6 *expression are shown for **(C) **ER^+ ^patients, **(D) **patients of LumA subtype, **(E) **patients of LumB subtype, and **(F) **ER^+^, tamoxifen-treated patients. The numbers in the parentheses equal patient numbers in each group, and the log rank *P *value is indicated at the bottom left of each figure.

Breast cancers were classified based on hormone-receptor status, estrogen receptor (ER^+/-^), or gene expression into intrinsic subtypes as described by Perou *et al. *[23]. Because these subclasses show differential survival, we analyzed the expression level of *JMJD6 *in each subtype annotated in the Super Cohort. As shown in Figure 2A, overall ER^- ^tumors had slightly but significantly elevated expression of *JMJD6 *(*P *< 0.001). The average *JMJD6 *expression differed across the intrinsic subtypes. *JMJD6 *expression was highest in Claudin-low tumors and basal subtypes, followed by HER2, whereas in the ER^+ ^group, the luminal B (LumB) expressed more *JMJD6 *than did luminal A (LumA) (*P *< 0.05) (Figure 2B). We examined the survival outcome of *JMJD6 *high- and low-expresser groups, determined by median separation across the Super Cohort, within each subtype. Because ER^- ^tumors had very high levels of *JMJD6 *expression, further separation based on *JMJD6 *levels and survival analysis bore no significance (Table 1). In ER^+ ^tumors, high *JMJD6 *expressers had poorer outcome than low expressers (Figure 2C; *P *< 0.001), and this persisted on subdivision of ER^+ ^into luminal subclasses (Figure 2D and 2E). Further, in the tamoxifen-treated samples, high *JMJD6 *expressers had worse outcome. However, whether *JMJD6 *predicts endocrine failure must be studied further. These data are summarized in Table 1. Other than total cohort median-based separation of patients, we also examined the relation of *JMJD6 *levels and prognosis by quartile assignment of *JMJD6 *expression in each subtype independently. With this method, we observed that extremely high expression of *JMJD6 *(upper quartile) significantly led to worse survival outcomes in luminal A and luminal B subtypes (*P *< 0.05) (see Additional file 3, Figure S1). Therefore, we conclude that *JMJD6 *is a marker for tumor aggressiveness and maybe is prognostic of poor outcome in ER^+ ^breast cancer.

### JMJD6 protein associates with high-grade and ER^- ^tumors

**Figure 3:**
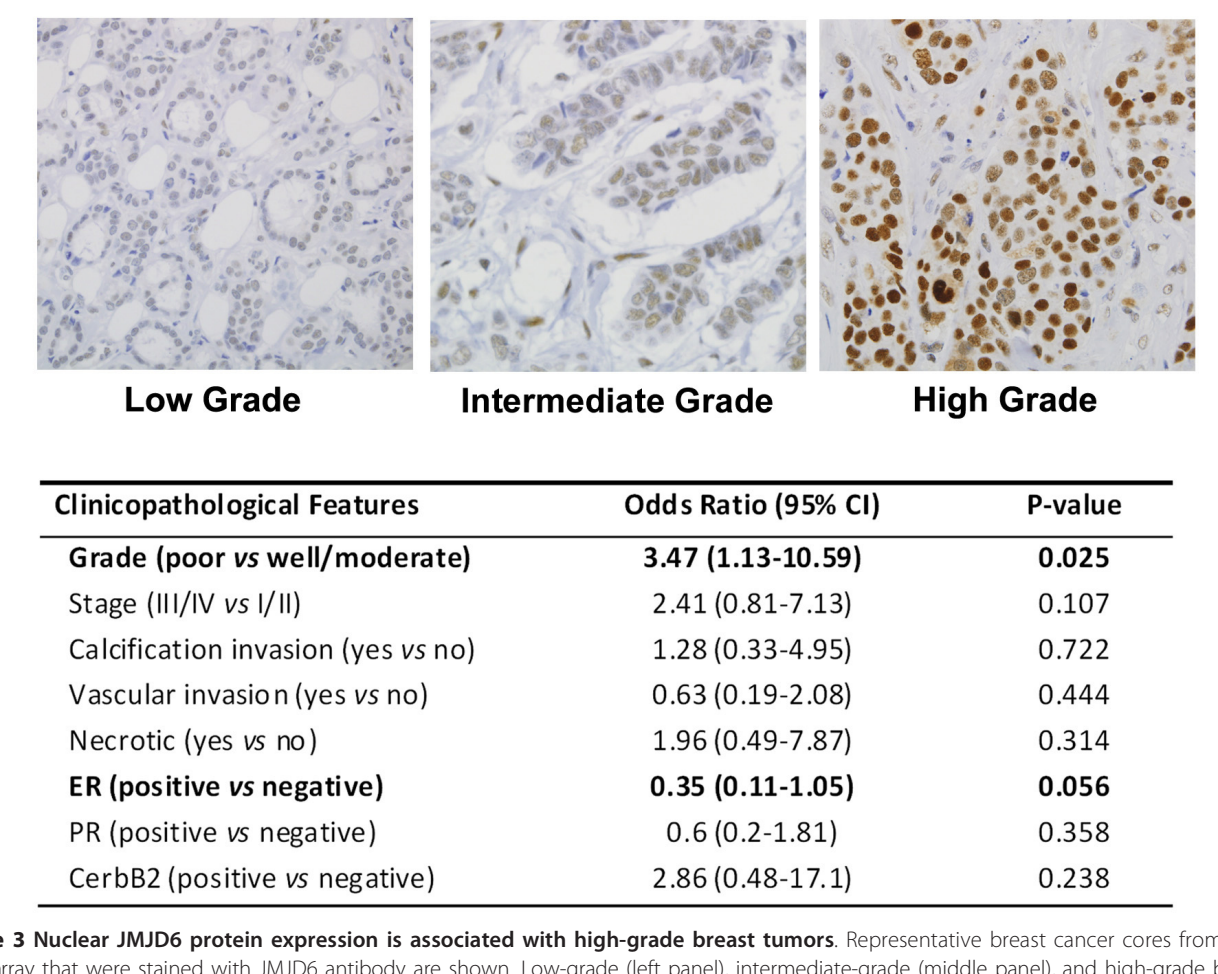
**Nuclear JMJD6 protein expression is associated with high-grade breast tumors**. Representative breast cancer cores from tissue microarray that were stained with JMJD6 antibody are shown. Low-grade (left panel), intermediate-grade (middle panel), and high-grade breast tumors (right panel) are shown (magnification, ×60). The table in the figure shows univariate analysis of JMJD6 and clinical parameters.

To study the distribution of JMJD6 protein levels, we immunohistochemically (IHC) stained an 81-breast tumor tissue array by using a JMJD6-specific antibody (Figure 3A). MCF-7-J1 OE and JMJD6 siRNA-treated cells were used to confirm the specificity of the antibody (see Additional file 4, Figure S2). As shown, JMJD6 was present predominantly in the nuclei of breast tumor cells, with increased staining evident in higher grades of breast tumors. Univariate analysis clearly identified an association between high JMJD6 staining, high grade, and ER negativity (Figure 3B). Similar to the RNA-expression data, JMJD6 protein was highly expressed in more-aggressive and advanced tumors. However, because of the low sample size, our analysis could not explore whether JMJD6 correlates with outcome in luminal tumors. Nonetheless, JMJD6 appears to be a relevant marker in breast cancer pathogenesis.

### JMJD6 increases proliferation in MCF-7 cells

**Figure 4:**
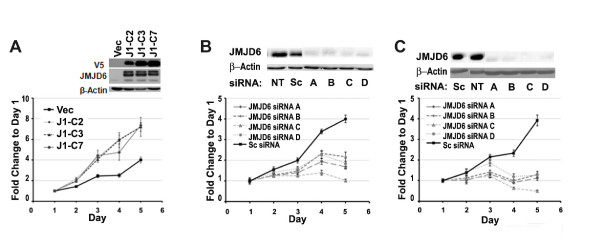
**Expression of JMJD6 enhances proliferation**. **(A) **WST-1 assays using MCF-7-J1-OE clones (J1-C2, J1-C3, and J1-C7) showed increased proliferation over vector control cells (Vec). SiRNA-mediated knockdown of JMJD6 and resultant decrease in proliferation is shown in **(B) **MCF-7 and MDA-MB231. Four individual siRNAs (A, B, C, and D) were used: Sc, scrambled siRNA; and NT, non-transfected cells served as control. Inset in each panel shows protein levels of JMJD6 after siRNA transfection. β-actin immunoblots show equal protein loading in all lanes.

Because the *JMJD6 *expression profile was similar to the proliferative gene cluster, as characterized by Perou *et al. *[14,24], we posited that perturbation of its levels may affect cell proliferation (Figure 2A and 2B). We studied JMJD6 expression in a panel of five breast cancer cell lines (see Additional file 5, Figure S3A). With the exception of MCF-7 cells, JMJD6 was highly expressed in breast cancer cell lines. We used a lentiviral-based cloning system to infect MCF-7 cells with V5-tagged JMJD6 and achieved stable expression of JMJD6 (MCF-7 J1-OE). As a pool, these cells showed increased proliferation (see Additional file 6, Figure S4), and this was confirmed in individual clones, J1-C2, J1-C3, and J1-C7 (Figure 4A). Over a period of 5 days, MCF-7-J1-OE clones (J1-C2, J1-C3, and J1-C7) showed higher proliferation as early as day 2 and continued to grow faster as compared with empty vector control cells (Vec) (Figure 4A). Next we silenced *JMJD6 *expression in all five cell lines by using a panel of siRNAs and assayed their proliferation. We achieved 80% to 100% silencing of JMJD6 protein, depending on the basal, endogenous expression level of JMJD6 (see Additional file 5, Figure S3A). Four different siRNAs resulted in profound loss of proliferation in MCF-7 and MDA MB231 cells (Figure 4B and 4C). This decrease was not due to increased apoptosis, because higher levels of cleaved PARP were not evident in the JMJD6 knockdown cells (see Additional file 5, Figure S3B). Similar to MCF7 and MDA-MB231, siRNA treatment decreased proliferation in the remaining cell lines: BT-549, CAMA-1, and T47D (see Additional file 5, Figure S3C). Together, these results suggest that high levels of JMJD6 lead to increased cell proliferation. To determine whether further induction of JMJD6 in cells with intrinsically higher JMJD6 levels shows an additive increase in cell proliferation, we generated MB231-J1-OE, T47D-J1-OE, and CAMA-J1-OE cells (see Additional file 7, Figure S5). However, an increased dosage of JMJD6 did not further augment cell growth

### JMJD6 enhances scattering and motility

**Figure 5:**
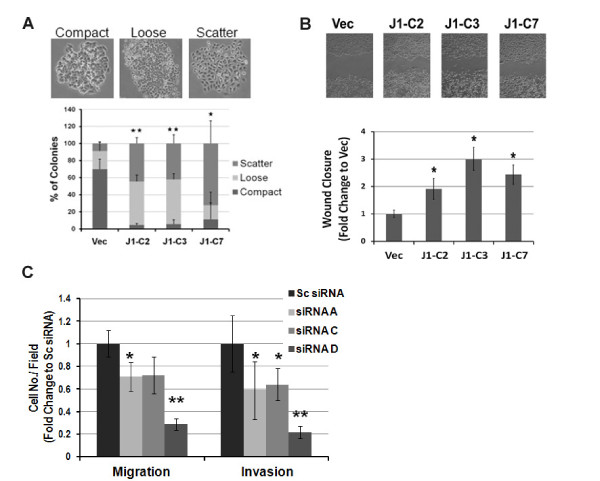
**JMJD6 promotes motility in breast cancer cell lines**. **(A) **Scatter phenotype was scored based on three levels of scattering (compact, loose, scatter) (upper panel). Bar graphs represent quantification of scattering of colonies by using the Student *t *test (lower panel). MCF-7 J1 OE clones had a higher percentage of scattered or loosely scattered clones than did the control Vec cells. **(B) **Wound-healing assay showed that MCF-7 J1 clones displayed higher motility in comparison to Vec (upper panel); quantification of wound closure as a ratio to the Vec is shown in the lower panel. *P ≤ 0.05 by Student *t *test in comparison to Vec control. **(C) **Boyden-chamber assay results of MDA-MB231 cells with JMJD6 siRNA-mediated knockdown were normalized to fold change in proliferation at Day 2 of WST-1 measurement. Two of three siRNA knockdowns of JMJD6 showed significantly decreased motility, and all siRNA knockdowns of JMJD6 showed decreased invasiveness. *P ≤ 0.05; **P ≤ 0.001 by Student's t- test when compared with Sc siRNA.

Our *in silico *analysis and IHC data showed that *JMJD6 *is associated with aggressive advanced disease. We next studied whether this protein could influence cell motility and invasion by using cell-scatter, wound-healing, and Boyden chamber assays. The motility and invasion assays were performed within 24 hours to minimize secondary effects of cell proliferation. For the scattering phenotype, we used a counting system previously described by Shtutman *et al. *[19] to evaluate the degree of scattering (Figure 5A). All three MCF-7-J1-OE clones showed an increase in scattering as compared with Vec cells. An increase in scattering often implies a gain in motility or epithelial-mesenchymal transformation (EMT). Therefore, we assayed loss of E-cadherin and gain in vimentin as markers for EMT. Immunoblots showed that MCF-7J1-OE clones maintained E-cad expression (see Additional file 8, Figure S6), suggesting that the scattering is probably encouraged by motility. Indeed, in a wound-healing assay, MCF-7-J1-OE clones closed the wound much faster than did Vec cells (Figure 5B). Because increased cell number due to increased proliferation can mislead interpretation of wound-healing data; we performed these assays in the presence of mitomycin C, an inhibitor of cell division. Despite a gain in motility, we were unable to demonstrate an increase in invasive behavior in MCF-7-J1-OE cells (data not shown). However, in MDA-MB231, a highly motile and invasive cell line, knockdown of JMJD6 significantly attenuated both properties (Figure 5C). To negate the contribution of decreased proliferation in this assay, we normalized these results to the proliferation changes observed on day 2 (Figure 5C). However, the cells maintained loss in motility and invasion, showing that this property was independent of proliferation defects. Together, these data imply that JMJD6 is not a "driver" of cell invasion but may augment cell movement.

### *JMJD6 *expression inversely correlates with *TGF-β2 *

**Figure 6:**
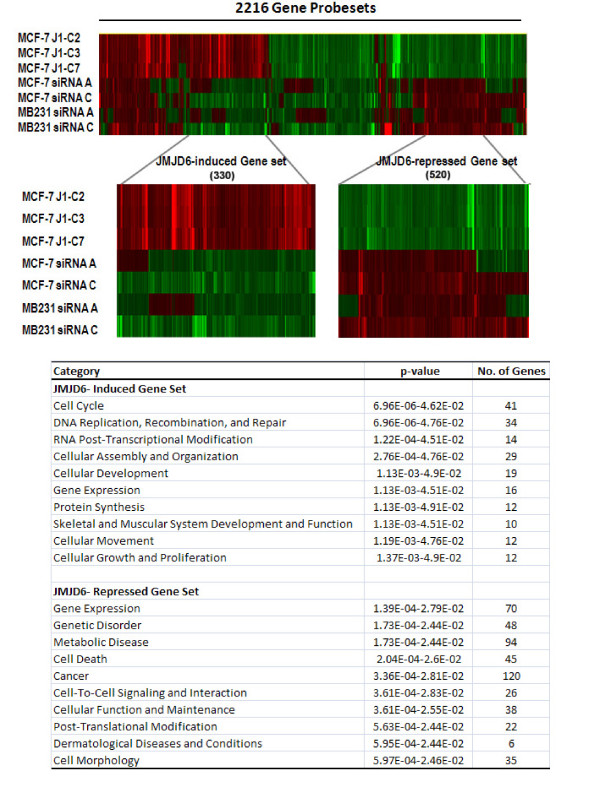
**IPA analysis of JMJD6-regulated genes showed enrichment in cell-cycle function**. **(A) **The heatmap (top panel) shows that hierarchic clustering of the genes changed after siRNA knockdown and overexpression of *JMJD6*. The sample labels are on the top of the heat map. Two clusters representing JMJD6-induced and JMJD6-repressed genes were selected (middle heat-map panel) and subjected to IPA analysis. **(B) **Table shows a list of the top 10 functions that are enriched in cells with altered levels of JMJD6.

**Figure 7:**
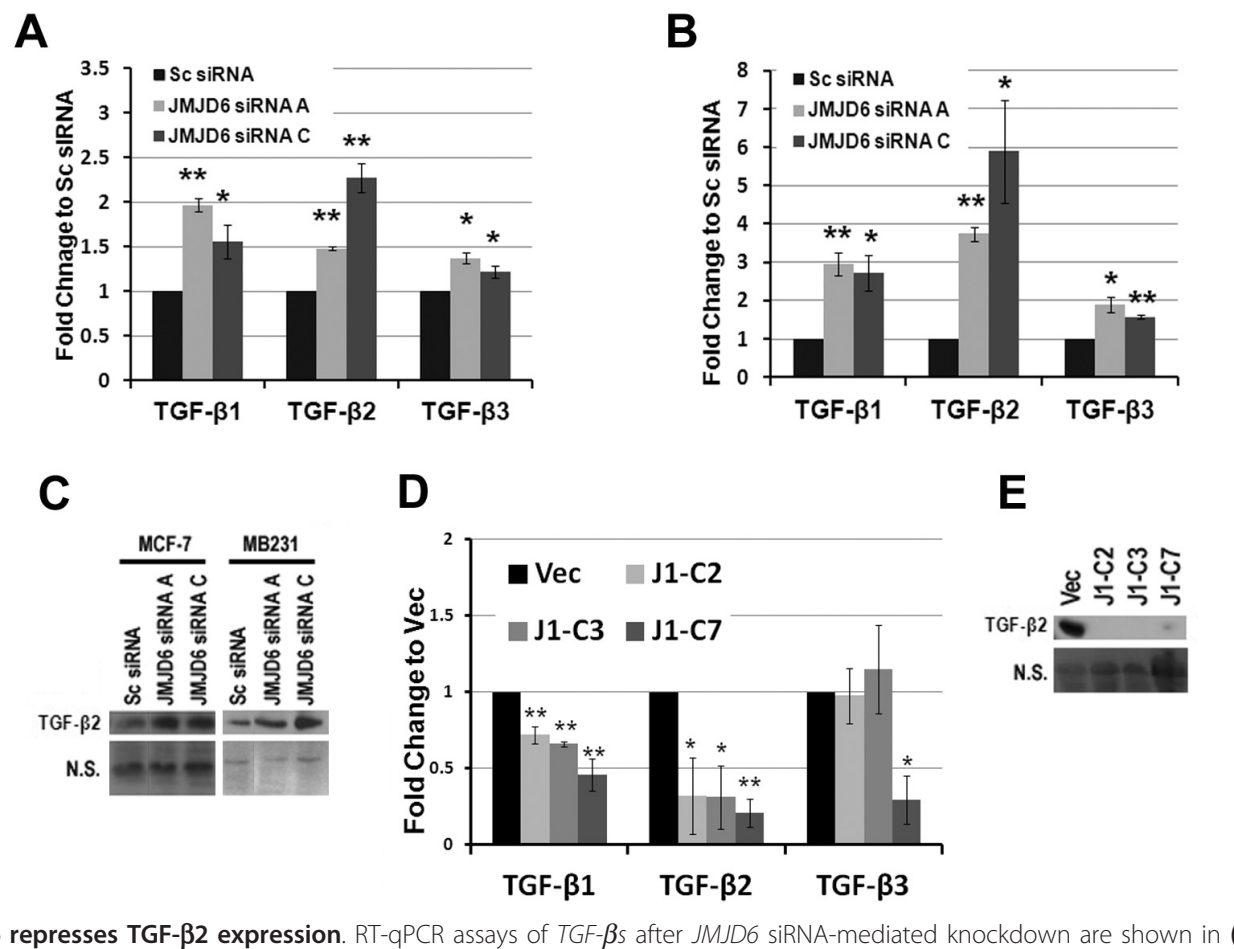
**JMJD6 represses TGF-β2 expression**. RT-qPCR assays of *TGF-βs *after *JMJD6 *siRNA-mediated knockdown are shown in **(A) **MCF-7 and **(B) **MDA-MB231. Consistently, T*GF-β2 *and *TGF-β1 *mRNA levels were upregulated on JMJD6 knockdown. **(C) **Immunoblots showed that the amount of secreted TGF-β2 protein in conditioned media was higher after *JMJD6 *siRNA-mediated knockdown in MCF-7 and MDA-MB231. **(D) **RT-qPCR assay showed that MCF7 J1 OE clones had a lower level of *TGF-β1 *and *TGF-β2 *transcripts than did the Vec cells. **(E) **Western blot showed a dramatic decrease in secreted TGF-β2 protein, in the MCF-7 J1 clones, as compared with the Vec cells. NS, a Ponceau-stained blot of the conditioned media to demonstrate protein loading. Student's t*- *test was performed by using scrambled siRNA versus JMJD6 siRNA and Vec cells versus MCF-7J1 clones. *P ≤ 0.05; **P ≤ 0.005.

To determine the pathways involved in JMJD6-induced proliferation, we performed microarray analysis by using the MCF-7-J1-OE clones and *JMJD6 *siRNA-treated MCF-7 and MDA-MB231 cells. We selected 2,216 overlapping probe sets that were commonly and appropriately regulated in all three settings: MCF-7-J1-OE, MCF-7 siRNA, and MDA-MB231 siRNA (see Additional file 9, Table S3). Clusters of genes regulated in the opposite directions in JMJD6 overexpression and JMJD6 knockdown were denoted JMJD6-induced and JMJD6-repressed gene sets, respectively (Figure 6). We analyzed these two gene sets with Ingenuity Pathway Analysis [20]. In accordance with our cell-based assays, in the JMJD6-induced gene set, the top enriched functions were cell-cycle and DNA replication, followed by cellular assembly and cellular movement (Figure 6; see Additional file 10, Table S4). In the JMJD6-repressed subset, a large number of genes were related to cancer function (120) (Figure 6). Among the associated gene functions, we observed a significant downregulation of *TGF-β *isoforms, the classic cell-cycle inhibitors [25-27] (see Additional file 10, Table S4), which was further confirmed by using quantitative RT-PCR. Loss of JMJD6 increased *TGF-β1 *and *TGF-β2 *expression, whereas *TGF-β3 *was unaffected in both MCF-7 and MDA-MB231 (Figure 7A and 7B). Protein analysis suggested that both JMJD6 siRNAs induced a high level of TGF-β2 secretion in the two cell lines (Figure 7C). In contrast, in MCF-7-J1-OE cells, a significant decrease in *TGF-β2 *mRNA and protein expression was observed (Figure 7D and 7E). At the protein level, we failed to detect changes in TGF-β1 and TGF-β3, although both isoforms were expressed (data not shown). Therefore, of the three isoforms, TGF-β2 probably was more engaged in JMJD6-mediated cellular proliferation.

### JMJD6 possibly engages multiple pathways to increase cell proliferation

JMJD6 suppressed *TGF-β2 *expression and cell proliferation in both MCF-7 and MDA-MB 231 cells. However, TGF-β2 mediates cell-cycle arrest only in MCF-7 cells, but not in MDA-MB 231, T47D, and CAMA-1 cells [28-30]. Therefore, TGF-β2 may not be the mediator of cell-cycle arrest in the latter three cell lines, even though MDA-MB231s are responsive to TGF-β in terms of SMAD phosphorylation and TGF-β-mediated transcriptional changes [31]. To test the functionality of TGF- β2, we did the following: (a) assessed Smad2/3 phosphorylation levels in both cells, (b) neutralized TGF-β2 by using a TGF-β type I receptor inhibitor SB431542 and assayed Smad phosphorylation and cell proliferation in the absence of JMJD6, and (c) treated MCF-7-J1OE cells with recombinant TGF- β2.

**Figure 8:**
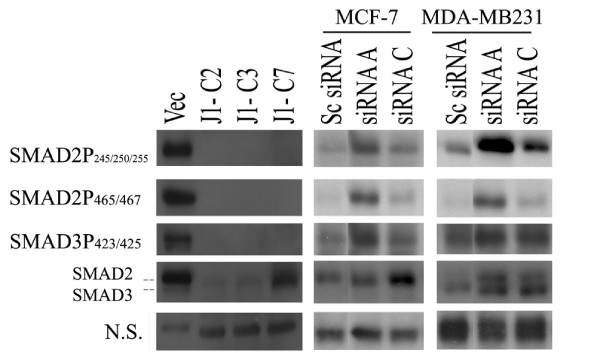
**JMJD6 expression affects SMAD phosphorylation**. Immunoblots show that the levels of total SMAD2/3, phosphorylated SMAD2, and phosphorylated SMAD3 are decreased in MCF-7 J1-OE clones and increased in JMJD6 siRNA-mediated knockdown in MCF-7 and MDA-MB231. The numbers next to the SMAD2P/3P denote the expected sites of phosphorylation detected by the antibodies. NS, nonspecific bands from the blots, which indicate equal loading of the protein lysates.

**Figure 9:**
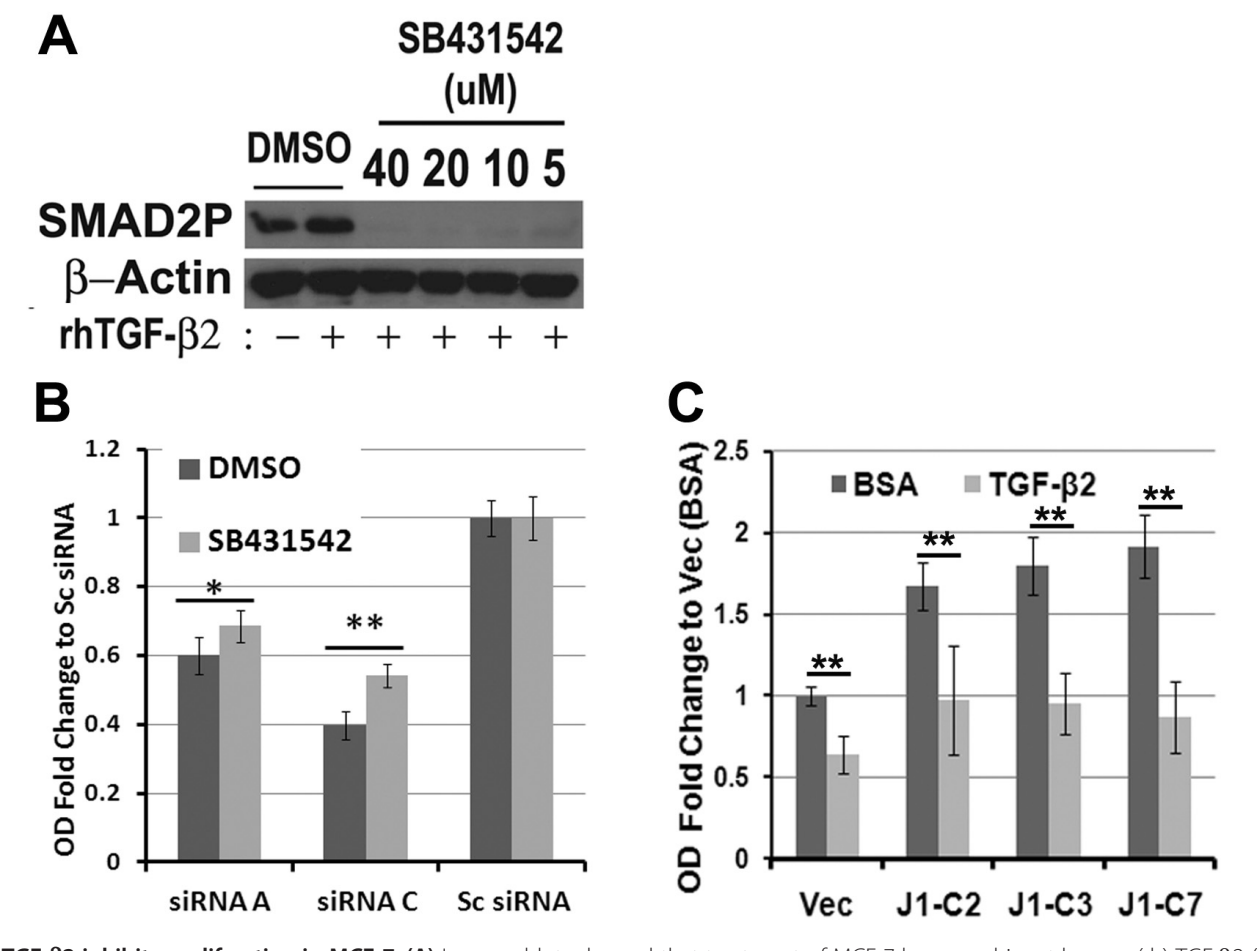
**TGF-β2 inhibits proliferation in MCF-7**. **(A) **Immunoblots showed that treatment of MCF-7 by recombinant human (rh) TGF-β2 (5 ng/ml) resulted in enhanced Smad2 phosphorylation, which could be effectively nullified by SB431542. **(B) **Treatment of JMJD6 siRNA-transfected MCF-7 cells with SB431542 (10 μ*M*) resulted in rescue of the proliferation defect. Ratio of OD at Day 3 to Day 1 in JMJD6 knockdown cells was normalized to the scrambled siRNA control. *P ≤ 0.05; **P ≤ 0.005 between DMSO and SB431542 treatments. **(C) **Treatment of MCF-7 J1-OE and Vec cells with rhTGF-β2 resulted in decreased proliferation, as measured by WST-1 assay. **P ≤ 0.001 between BSA and rhTGF-β2 treatments.

By treatment of recombinant TGF-β2 on MCF-7 and MDA-MB231, we confirmed that both cell lines responded with increased SMAD2 phosphorylation, which suggests intact TGF-β receptor signaling (Figure 8; see Additional file 11, Figure S7A). Similarly, an increase in SMAD2 and SMAD3 phosphorylation with *JMJD6 *siRNA treatment was seen in both MCF-7 and MDA-MB231, as opposed to a decrease observed in the MCF-7 J1 OE clones (Figure 8). Interestingly, basal SMAD levels themselves were affected, which parallels the transcript-level change detected in our expression array (Figure 6). When we inhibited SMAD phosphorylation by using an Alk-5 receptor inhibitor (SB431542), we observed a significantly higher rescue of proliferation in MCF-7 *JMJD6 *knockdown cells (Figure 9A and 9B). Because MDA-MB 231 cells are refractory to TGF-β2-mediated cell-cycle inhibition, SB431542 should have no effect on MDA-MB231 growth suppression. Accordingly, the suppressed proliferation could not be rescued by the Alk-5 receptor inhibitor treatment in this cell type (see Additional file 11, Figure S7).

Last, treatment of MCF-7 J1-OE cells with recombinant TGF-β2 repressed proliferation in both Vec and J1-OE cells, suggesting that MCF-7 retained sensitivity to TGF-β2-mediated growth regulation despite increased JMJD6 levels (Figure 9C). Therefore, JMJD6 did not confer unresponsiveness to this growth factor, but probably mediated its effect via repression of gene expression. Together these data suggest that in MCF-7 cells, JMJD6 mediates cell-cycle regulation, in part by suppressing *TGF-β2*.

**Figure 10:**
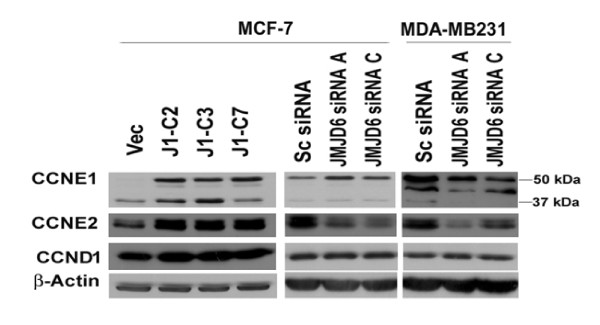
**Decrease of cyclin E2 protein level in JMJD6 knockdown**. Western blot detection of G_1 _cell-cycle cyclins is shown. A decrease in cyclin E2 (CCNE2) level with decrease in JMJD6 levels was evident in MCF-7 and MDA-MB231. Cyclin E1 (CCNE1) and cyclin D1 (CCND1) displayed an inconsistent change on JMJD6 siRNA transfection in both MCF-7 and MDA-MB231 cells.

Secretion of TGF-β2 cannot explain the growth arrest in *JMJD6 *siRNA-treated MDA MB 231 cells. Scanning of microarray data revealed that JMJD6 changed the levels of G_1 _cyclins: *cyclins E1, E2*, and *D *at the RNA level. In MDA MB 231 cells, we confirmed a loss in cyclin E2 protein expression but not E1 or cyclin D in JMJD6 siRNA-containing cells (Figure 10). Interestingly, cyclin E1/E2 was decreased in MCF-7 *JMJD6 *siRNA-treated cells and induced in MCF-7-J1 OE cells and (Figure 10). Together these data indicate that in both cells, JMJD6 mediated suppression of *TGF-β2*. In MCF-7 cells, the proliferation is controlled by both TGF-β2 and cyclin E regulation, but in MDA 231 cells, the cyclin E regulation may contribute to this phenotype, rather than TGF-β2 signaling. Other pathways that operate in MDA-MB 231 cells must be explored further.

### Clinical data for *JMJD6 *and *TGF-β2 *

**Figure 11:**
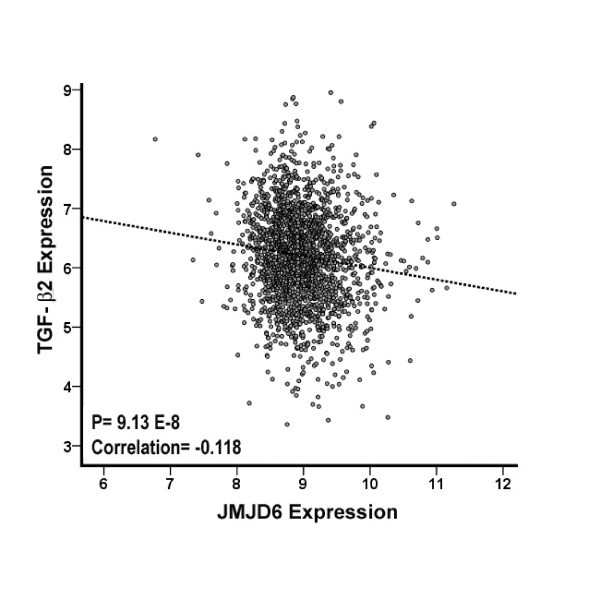
**Clinical association of *JMJD6 *with *TGF-β2***. Pearson correlation of clinical expression of *JMJD6 *with *TGF-β2 *suggests a negative correlation with *TGF-β2 *(*P *= 9.13 × 10^-8^). Scatterplot shows the normalized *JMJD6 *expression of each patient sample with the corresponding normalized *TGF-β2 *expression in the clinical dataset of 2,034 breast cancer patients.

Finally, by using expression data from the clinical cohorts, we observed a marginal but significant inverse correlation *of JMJD6 *with *TGF-β2 *(Pearson correlation value, -0.118; *P *value = 9.13 × 10^-8^) (Figure 11). This suggests that this *TGF-β2 *mechanism observed *in vitro *could be extrapolated to patient data and that the MCF-7-OE cells could serve as a model system to explore the pathways involved in further detail.

## Discussion

Through microarray informatics, we identified *JMJD6 *as a candidate oncogene associated with poor patient prognosis. Consistent with its potential as a marker of breast cancer aggressiveness, *JMJD6 *was expressed at highest levels in ER^-^, basal, claudin-low, *HER2*-enriched, and Luminal B tumor subtypes, which are known to be clinically associated with poor patient outcomes. Most important, in the less-aggressive ER^+ ^subtypes (for example, Luminal A), high levels of *JMJD6 *could predict poor outcome and possibly resistance to tamoxifen monotherapy. Protein analysis by using tissue microarray confirmed that high *JMJD6 *expression is consistently associated with ER^- ^disease, higher grade, and advanced stage. These data demonstrate for the first time that JMJD6 is a relevant prognostic biomarker in breast cancer.

By phenotypic and functional analysis, we found that *JMJD6 *induced an increase in cell scattering and increased the rate of wound closure in MCF-7 cells, but did not confer an invasive phenotype. In invasive cells like MDA-MB231 and BT-549 with high expression of *JMJD6*, siRNA-mediated suppression led to loss of both motility and invasiveness properties. Because forced *JMJD6 *expression did not confer invasiveness, this apparent loss of invasiveness most likely follows a loss in motility. Nonetheless, JMJD6 may have a critical role in epithelial cell movement, and we showed that this property is not restricted to endothelial cells [7]. However, this appears to be a minor function of JMJD6, because its exogenous expression did not induce epithelial-to-mesenchymal transition (EMT) (that is, we did not observe a loss or gain in E-cadherin or vimentin expression, respectively, in MCF-7 J1-OE cells).

The most prominent effect of JMJD6 perturbation was on cell proliferation. SiRNA-mediated attenuation of JMJD6 in multiple breast cancer cells led to a decrease in cell proliferation without activation of cellular apoptosis, whereas forced expression in MCF-7 resulted in a massive increase in cell proliferation. This finding is consistent with the observation that JMJD6-knockout mice are small and show growth retardation [3]. The role of JMJD6 in proliferation is further substantiated by our microarray analysis, which revealed that modulation of JMJD6 expression significantly affected the expression levels of a number of cell cycle-associated genes (see Additional file 10, Table S4). Although *JMJD6 *is thought to mediate splicing by physical interaction with U2AF65, we obtained very little evidence for alternate transcripts in both cell lines. Conversely, we documented a large number of transcriptional changes in J1-OE clones and JMJD6 knockdowns.

The TGF-β pathway, particularly TGF-β2, was downregulated when JMJD6 was overexpressed and induced when JMJD6 was depleted. We validated the inverse relation between *JMJD6 *and *TGF-β2 *at both RNA and protein levels in these cellular systems. We extended this observation to clinical samples and observed an inverse correlation between *JMJD6 *and *TGF-β2 *in our breast cancer cohorts. Such antagonistic roles of JMJD6 and TGF-β2 have a precedent in eye development. One of the distinctive developmental defects of JMJD6-knockout mice is the thinning of the retinal layer [3]. Coincidentally, the knockout of TGF-β2 results in the hyperplasia of the neuroblastic layer of the retina [32]. These data suggest that JMJD6 and TGF-β2 may be functionally linked in cell-cycle regulation and in development.

Canonically, TGF-βs are known to exert antiproliferative effects by upregulating cyclin-dependent kinase inhibitors, p15 and p21, and/or downregulation of CDK2 and cyclin E activity [26,27,33-35]. In both MCF-7 and MDA-MB 231 transfected with *JMJD6 *siRNA, we observed suppression of cyclin E expression, and an increase in cyclin E was evident when *JMJD6 *was overexpressed in MCF-7 cells. Therefore, in both cell lines, JMJD6 may exert a proliferative effect through a direct increase in cyclin E protein levels, or this increase may be a consequence of JMJD6-mediated suppression of TGF-β2. In both MCF-7 and MDA-MB231, the downstream effectors, Smads, were phosphorylated on JMJD6 siRNA-mediated secretion of TGF-β2. Specifically, in MCF-7, inhibition of TGF-β activity by using a TGF-β type I receptor (Alk5) inhibitor resulted in the loss of Smad phosphorylation, rescue of arrested cells, and continuation of proliferation (Figure 9). The Alk5 inhibitor did not reverse cell-cycle arrest in MDA-MB 231 cells because they are refractory to the antiproliferative effect of TGF-β, suggesting that the JMJD6-cyclin E axis may function independent of TGF-β2 in these cells. Intriguingly, cyclin E is associated with poor prognosis, chromosomal instability, and trastuzumab resistance, suggesting that the long-term dysregulation of cell-cycle mediators by JMJD6 may affect more than just proliferation [36-38].

Our initial microarray analysis selected genes that were commonly regulated by JMJD6 in both MCF-7 and MDA-MB 231 cells. To elucidate further the mechanisms behind *JMJD6 *siRNA-mediated growth suppression in MDA-MB 231 cells, we reanalyzed gene-expression changes unique to MDA-MB 231 cells alone. We found 1,864 probesets, with significant enrichment of genes involved in cellular death, growth and proliferation, and cell movement (*P *< 0.01; see Additional file 12, Figure S8). Close inspection showed at least 28 individual genes in this proliferation gene cassette (see Additional file 13, Table S5); however, no connectivity or compelling pathway emerged that could be investigated further.

## Conclusion

In summary, our data indicate that *JMJD6 *has conserved functions and often affects similar pathways in a congruent manner across multiple cell types and at a gene-expression and phenotypic level. JMJD6 has ability to promote cancer cell proliferation and motility, which in turn may augment cancer virulence *in vivo*. In the clinic, JMJD6 associates with advanced grade, an aggressive phenotype, and serves as a marker of poor prognosis in breast cancer. We propose that JMJD6 staining (with IHC) may serve a dual purpose in the clinic: to predict poor outcome in patients, particularly in the ER^+ ^subtype, and to identify a patient subgroup wherein specific inhibitors of JMJD6 may facilitate the pathologic downstaging of tumors in the neoadjuvant setting.

## Abbreviations

DMEM: Dulbecco Modified Eagle Medium; DMFS: distant metastasis-free survival; ER: estrogen receptor; Flt1: vascular endothelial growth factor receptor 1; HER2: human epidermal growth factor receptor 2; IHC: immunohistochemistry; IPA: ingenuity pathway analysis; JMJD6: Jumonji domain-containing 6 protein; LumA: luminal A; LumB: luminal B; MCF-7-J1-OE: MCF-7 JMJD6 overexpression; PTDSR: phosphatidylserine receptor; RT-PCR: real-time polymerase chain reaction; SPINK1: serine protease inhibitor Kazal type 1; TGF-β: transforming growth factor-β; TMA: tissue microarray; U2AF65: U2 auxiliary factor 65.

## Competing interests

The authors declare that they have no competing interests.

## Authors' contributions

This study was designed and written by KVD, ETL, and YFL. YFL performed all experiments with assistance from XBC. Breast cohort analysis was done by LDM, YFL, and MAB. BP, CWO, and MST provided the NUH tissue arrays and performed and scored the immunostaining. All authors read and approved the final manuscript.

## Supplementary Material

Additional file 1 **Table S1. QPCR Primers**. QPCR primer sequences used in the RT-PCR. Click here for file

Additional file 2 **Table S2. Microarray datasets**. List of clinical microarray datasets used in the survival analysis of *JMJD6 *and their general information, including database source and literature references. Click here for file

Additional file 3 **Figure S1. Upper-quartile expression of *JMJD6 *is associated with poor survival in LumA and LumB subtypes**. Kaplan-Meier survival curves were generated based on quartile ranking of *JMJD6 *expression within the individual subtype. Representative images are shown for **(A) **LumA subtype and **(B) **LumB subtype. *Log rank *P *< 0.05; #log rank *P *< 0.001 between two quartile expression groups within the subtype. Click here for file

Additional file 4 **Figure S2. Standardization of JMJD6 immunohistochemistry**. IHC for JMJD6 was performed on wild-type (left panel), *JMJD6 *siRNA-mediated knockdown (middle panel), and JMJD6-overexpressing (right panel) MCF-7 cells. As shown in the figure, wild-type and *JMJD6 *siRNA cells showed low to negligible amounts of immunoreactivity, whereas expression of JMJD6 was the highest in MCF7-J1-OE cells. Click here for file

Additional file 5 **Figure S3. Protein expression and knockdown of JMJD6 in other cell lines. (A) **Western blot of different breast cancer cell lines suggests that JMJD6 protein expression was low in MCF-7, whereas MDA-MB231, T47D, CAMA-1, and BT-549 cells harbored substantial amounts of JMJD6 protein. **(B) **Level of apoptosis in cells transfected with *JMJD6 *siRNA was determined by using a PARP-cleavage assay. Bar chart shows that the percentage of cells with cleaved PARP was similar in both *JMJD6 *siRNA and scrambled siRNA-transfected MCF-7 cells. **(C) **Decreased proliferation was observed in BT-549, CAMA-1, and T47D when JMJD6 was knocked down by three individual JMJD6-specific siRNAs after 4 days of plating, as compared with the scrambled siRNA control (Sc siRNA); *P *≤ 0.005. Error bars represent standard deviation. Click here for file

Additional file 6 **Figure S4. Pooled population of MCF-7 J1-OE cells showed increased proliferation and decreased TGF-β2 levels. (A) **WST-1 assay using MCF-7 J1-OE pooled population before clonal selection showed increased proliferation over vector control cells (Vec). **(B) **Immunoblots of conditioned media showed decreased levels of secreted TGF-β2 in MCF-7 J1-OE pooled population as compared with Vec. NS, nonspecific bands from the same TGF-β2 antibody blot to indicate even total protein loading. Click here for file

Additional file 7 **Figure S5. Overexpression of JMJD6 in CAMA, T47D, and MDA-MB231 had no effect on proliferation**. Figures (top panel) show Western blot for V5-tagged JMJD6 clones expressed in **(A) **CAMA, **(B) **T47D, and **(C) **MDA-MB231, respectively, and β-actin as an internal control for loading. A through C (bottom) show that stable *JMJD6 *overexpression clones did not display increased proliferation as compared with the Vec control. Click here for file

Additional file 8 **Figure S6. MCF-7 J1-OE cells did not show changes in E-cadherin and vimentin levels**. Immunoblots showed that the expression of E-cadherin is similar in the MCF-7 J1-OE cells and Vec cells. Vimentin remained unexpressed in MCF-7 J1-OE cells. MDA-MB231 was used as a positive control for the vimentin antibody. Click here for file

Additional file 9 **Table S3. Microarray analysis**. List of genes that were differentially expressed by microarray analysis of MCF-7 J1-OE clones and JMJD6 siRNA knockdown cells. Click here for file

Additional file 10 **Table S4. JMJD6 and gene function**. Top ten functions enriched in JMJD6-induced and JMJD6-repressed gene sets by IPA functional analysis and the list of genes in each function. Click here for file

Additional file 11 **Figure S7. SB431542 does not rescue JMJD6 siRNA-mediated loss of proliferation in MDA-MB231. (A) **Immunoblots showed that treatment of rhTGF-β2 in MDA-MB231 resulted in enhanced SMAD2 phosphorylation, which could be nullified by SB431542 treatment. **(B) ***JMJD6 *siRNA-mediated decreased proliferation of MDA-MB231 could not be rescued by SB431542 (10 μ*M*) treatment, as assessed with WST-1 OD measurement. Click here for file

Additional file 12 **Figure S8. Functional annotation of differentially expressed genes unique to MDA-MB 231 cells transfected with *JMJD6 *siRNA**. Bar chart shows IPA functional annotation analysis of differentially expressed genes in MDA-MB231 *JMJD6 *siRNA-mediated knockdown showed significant enrichment of genes involved in cellular death, growth, and movement. Log *P*-value on the X-axis is Fisher Exact test on the overlap of our gene list and the functional category (Additional File 13, Table S5). Click here for file

Additional file 13 **Table S5. "Proliferation" genes differentially expressed in MDA-MB231**. List of genes found in subcategories under the functional annotation for cellular growth and proliferation that were significantly enriched (*P *< 0.05). The *P *value is from the Fisher Exact test for the overlap of our dataset and the function. The predicted activation state is stated for significant direction of change (Z score, ≤-2). Click here for file
