# Erratum to: JMJD6 is a driver of cellular proliferation and motility and a marker of poor prognosis in breast cancer

**DOI:** 10.1186/s13058-017-0830-9

**Published:** 2017-03-28

**Authors:** Yi Fang Lee, Lance David Miller, Xiu Bin Chan, Michael A. Black, Brendan Pang, Chee Wee Ong, Manuel Salto-Tellez, Edison T. Liu, Kartiki V. Desai

**Affiliations:** 1 Clearbridge BioMedics Private Ltd, 81 Science Park Drive, Singapore, Singapore; 2 Department of Cancer Biology, Wake Forest University School of Medicine, Winston-Salem, NC 27157 USA; 3 Department of Pathology, National University Health System and National University of Singapore, 5 Lower Kent Ridge Road, Singapore, 119074 Singapore; 4 Centre for Cancer Research and Cell Biology, Queen’s University Belfast, Belfast, UK; 5 Cancer Science Institute, National University of Singapore, 28 Medical Drive, Singapore, 117456 Singapore; 6 Department of Biochemistry, Otago School of Medical Sciences, University of Otago, 710 Cumberland Street, Dunedin, 9054 New Zealand; 7 The Jackson Laboratory, Bar Harbor, ME 04609 USA; 8 National Institute of Biomedical Genomics, 2 Netaji Subash Sanatorium (T.B. Hospital), Kalyani, 741251 India

## Erratum

**Main text:** After the publication of this work [1] an error was noticed in Fig. 8. In the SMAD2P465/467 panel, the images for MCF-7 and MDA MB 231 cells were accidentally duplicated. The corrected figure is shown below. We apologize for this error, which does not affect the findings or conclusions of the article.

**Fig. 8:**
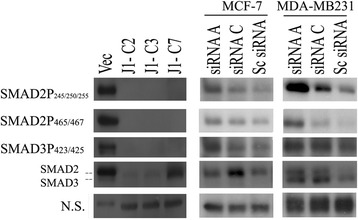
JMJD6 expression affects SMAD phosphorylation. Immunoblots show that the level of total SMAD2/3, phosphorylated SMAD2 and phosphorylated SMAD3 are decreased in MCF-7 J1-OE clones, and increased in JMJD6 siRNA-mediated knock-down in MCF-7 and MDA-MB231. The numbers next to the SMAD2P/3P denote the expected sites of phosphorylation detected by the antibodies. N.S. denotes non-specific bands from the blots which indicate equal loading of the protein lysates
