# Supplementary figures and images for: JMJD6 is a driver of cellular proliferation and motility and a marker of poor prognosis in breast cancer
Source: Breast Cancer Res. 2012 May 23;14(3):R85. doi: 10.1186/bcr3200 (PMC3446348; doi:10.1186/bcr3200)

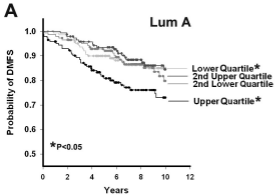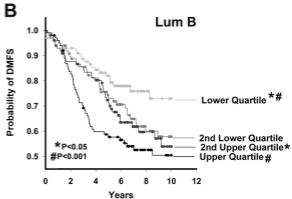

Supplement: Additional file 3 — Figure S1. Upper-quartile expression of JMJD6 is associated with poor survival in LumA and LumB subtypes. Kaplan-Meier survival curves were generated based on quartile ranking of JMJD6 expression within the individual subtype. Representative images are shown for (A) LumA subtype and (B) LumB subtype. *Log rank P < 0.05; #log rank P < 0.001 between two quartile expression groups within the subtype. [file bcr3200-S3.PDF]

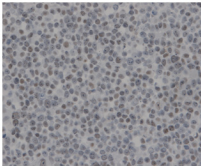

**Wildtype**

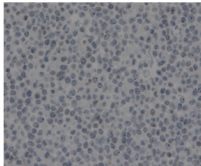

**JMJD6 Knock-down**

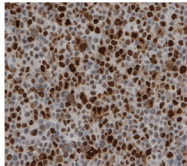

**JMJD6 Over-Expressed**

Supplement: Additional file 4 — Figure S2. Standardization of JMJD6 immunohistochemistry. IHC for JMJD6 was performed on wild-type (left panel), JMJD6 siRNA-mediated knockdown (middle panel), and JMJD6-overexpressing (right panel) MCF-7 cells. As shown in the figure, wild-type and JMJD6 siRNA cells showed low to negligible amounts of immunoreactivity, whereas expression of JMJD6 was the highest in MCF7-J1-OE cells. [file bcr3200-S4.PDF]

**A**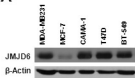**B**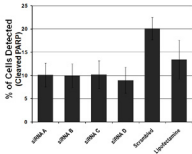**C**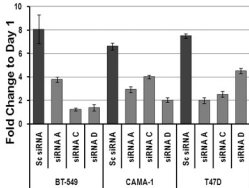

Supplement: Additional file 5 — Figure S3. Protein expression and knockdown of JMJD6 in other cell lines. (A) Western blot of different breast cancer cell lines suggests that JMJD6 protein expression was low in MCF-7, whereas MDA-MB231, T47D, CAMA-1, and BT-549 cells harbored substantial amounts of JMJD6 protein. (B) Level of apoptosis in cells transfected with JMJD6 siRNA was determined by using a PARP-cleavage assay. Bar chart shows that the percentage of cells with cleaved PARP was similar in both JMJD6 siRNA and scrambled siRNA-transfected MCF-7 cells. (C) Decreased proliferation was observed in BT-549, CAMA-1, and T47D when JMJD6 was knocked down by three individual JMJD6-specific siRNAs after 4 days of plating, as compared with the scrambled siRNA control (Sc siRNA); P ≤ 0.005. Error bars represent standard deviation. [file bcr3200-S5.PDF]

**A**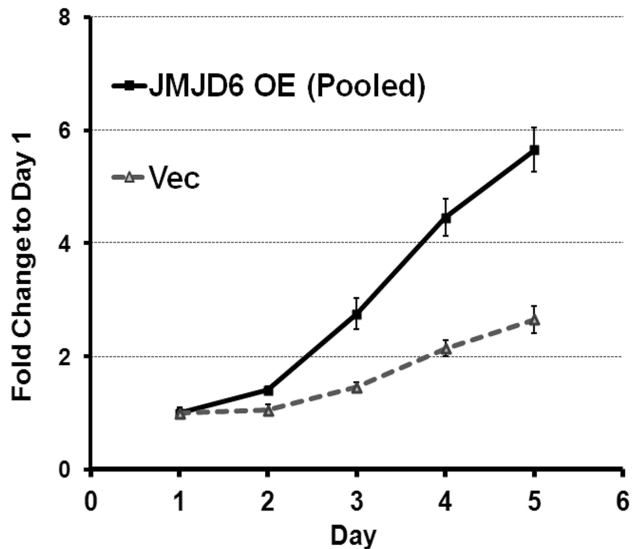**B**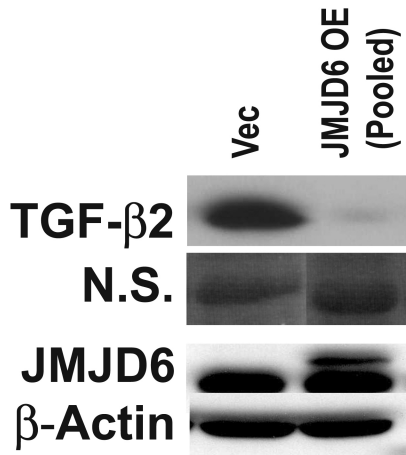

Supplement: Additional file 6 — Figure S4. Pooled population of MCF-7 J1-OE cells showed increased proliferation and decreased TGF-β2 levels. (A) WST-1 assay using MCF-7 J1-OE pooled population before clonal selection showed increased proliferation over vector control cells (Vec). (B) Immunoblots of conditioned media showed decreased levels of secreted TGF-β2 in MCF-7 J1-OE pooled population as compared with Vec. NS, nonspecific bands from the same TGF-β2 antibody blot to indicate even total protein loading. [file bcr3200-S6.PDF]

**A**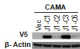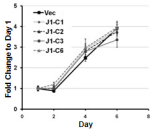**B**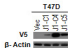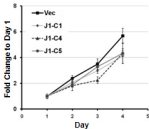**C**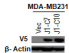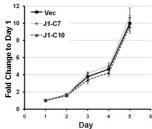

Supplement: Additional file 7 — Figure S5. Overexpression of JMJD6 in CAMA, T47D, and MDA-MB231 had no effect on proliferation. Figures (top panel) show Western blot for V5-tagged JMJD6 clones expressed in (A) CAMA, (B) T47D, and (C) MDA-MB231, respectively, and β-actin as an internal control for loading. A through C (bottom) show that stable JMJD6 overexpression clones did not display increased proliferation as compared with the Vec control. [file bcr3200-S7.PDF]

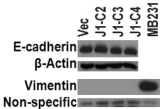

Supplement: Additional file 8 — Figure S6. MCF-7 J1-OE cells did not show changes in E-cadherin and vimentin levels. Immunoblots showed that the expression of E-cadherin is similar in the MCF-7 J1-OE cells and Vec cells. Vimentin remained unexpressed in MCF-7 J1-OE cells. MDA-MB231 was used as a positive control for the vimentin antibody. [file bcr3200-S8.PDF]

**A**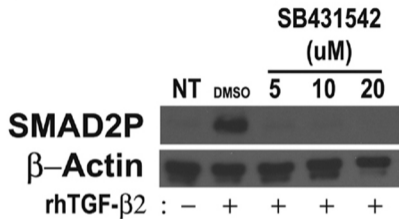**B**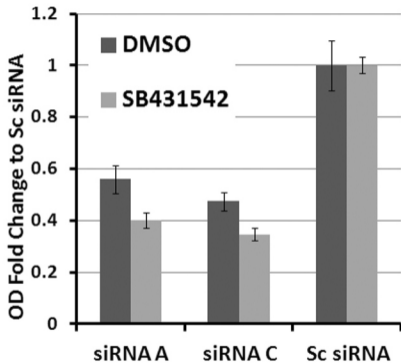

Supplement: Additional file 11 — Figure S7. SB431542 does not rescue JMJD6 siRNA-mediated loss of proliferation in MDA-MB231. (A) Immunoblots showed that treatment of rhTGF-β2 in MDA-MB231 resulted in enhanced SMAD2 phosphorylation, which could be nullified by SB431542 treatment. (B) JMJD6 siRNA-mediated decreased proliferation of MDA-MB231 could not be rescued by SB431542 (10 μM) treatment, as assessed with WST-1 OD measurement. [file bcr3200-S11.PDF]

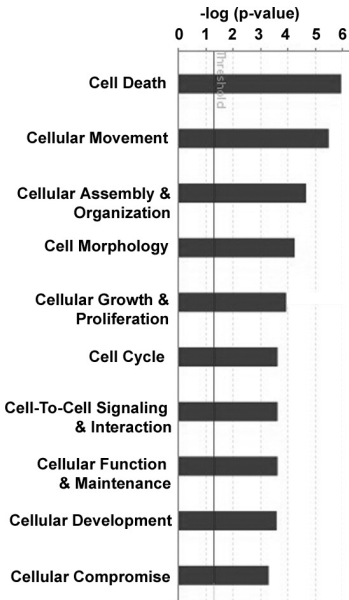

Supplement: Additional file 12 — Figure S8. Functional annotation of differentially expressed genes unique to MDA-MB 231 cells transfected with JMJD6 siRNA. Bar chart shows IPA functional annotation analysis of differentially expressed genes in MDA-MB231 JMJD6 siRNA-mediated knockdown showed significant enrichment of genes involved in cellular death, growth, and movement. Log P-value on the X-axis is Fisher Exact test on the overlap of our gene list and the functional category (Additional File 13, Table S5). [file bcr3200-S12.PDF]
